# Extensive scrofuloderma with recurrent tubercular lymphadenitis diagnosed as drug-resistant tuberculosis

**DOI:** 10.11604/pamj.2024.48.57.43896

**Published:** 2024-06-13

**Authors:** Jay Dinesh Bhanushali

**Affiliations:** 1 Department of Respiratory Medicine, Jawaharlal Nehru Medical College, Datta Meghe Institute of Higher Education and Research, Sawangi (Meghe), Wardha, Maharashtra, India

**Keywords:** Cutaneous tuberculosis, fistula, collar stud abscess, tubercular lymphadenitis

## Image in medicine

A 26-year-old male presented with a history of several painless lumps on the right side of the neck that began two years ago ulcerated about two months later, formed secreting fistulas, and then spontaneously healed after four months. Similar swellings appeared on the left side of the neck six months later. On examination, a 4cm x 3cm swelling was palpated in the left cervical region along with other small, matted lymph nodes, and a needle aspiration of the lymph node was performed. Chest X-rays showed no abnormalities. A clinical diagnosis of Tubercular Lymphadenitits was made. The pus aspirated from the lymph node tested positive for mycobacteria tuberculosis and rifampicin resistance using the cartridge-based nucleic acid amplification test (CB-NAAT, GeneXpert). The patient was started on a treatment regimen for drug-resistant tuberculosis, and the pus was sent for a second-line line probe assay (LPA) for further determination of drug resistance. Scrofuloderma is a skin infection near a tuberculosis focus. Its clinical presentation includes painless, slowly growing nodules beneath the skin, which eventually turn into ulcers and fistulas that discharge serous, purulent, or caseous material. It progresses gradually and may result in persistent discharge, chronic ulcers, scarring, or recovery. The cervical group of lymph nodes is the most frequently impacted, but there is also potential involvement of axillary, inguinal, pre-auricular, submandibular, and occipital lymph nodes as well. Histopathological examination typically reveals the presence of granuloma with a central core of caseous necrosis, and acid-fast bacilli may also be seen. This image represents the extensive nature of scrofuloderma and active tubercular lymphadenitis with antigravity aspiration of the lymph node.

**Figure 1:**
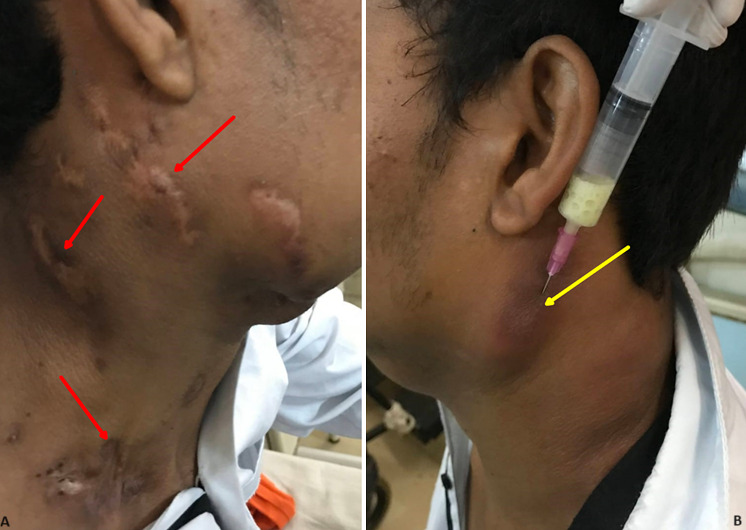
(A,B) extensive scrofuloderma (red arrows) with active tubercular lymphadenitis being aspirated (yellow arrow)

